# Three-Dimensional Numerical Simulation of Particle Focusing and Separation in Viscoelastic Fluids

**DOI:** 10.3390/mi11100908

**Published:** 2020-09-30

**Authors:** Chen Ni, Di Jiang

**Affiliations:** School of Mechanical and Electronic Engineering, Nanjing Forestry University, Nanjing 210037, China; nichen@njfu.edu.cn

**Keywords:** lattice Boltzmann method, particle focusing, particle separation, viscoelastic fluid, aspect ratios

## Abstract

Particle focusing and separation using viscoelastic microfluidic technology have attracted lots of attention in many applications. In this paper, a three-dimensional lattice Boltzmann method (LBM) coupled with the immersed boundary method (IBM) is employed to study the focusing and separation of particles in viscoelastic fluid. In this method, the viscoelastic fluid is simulated by the LBM with two sets of distribution functions and the fluid–particle interaction is calculated by the IBM. The performance of particle focusing under different microchannel aspect ratios (AR) is explored and the focusing equilibrium positions of the particles with various elasticity numbers and particle diameters are compared to illustrate the mechanism of particle focusing and separation in viscoelastic fluids. The results indicate that, for particle focusing in the square channel (AR = 1), the centerline single focusing becomes a bistable focusing at the centerline and corners as *El* increases. In the rectangular channels (AR < 1), particles with different diameters have different equilibrium positions. The equilibrium position of large particles is closer to the wall, and large particles have a faster lateral migration speed and few large particles migrate towards the channel center. Compared with the square channel, the rectangular channel is a better design for particle separation.

## 1. Introduction

Particle focusing is essential for particle counting and detection [1]. In addition, particle separation from mixed samples is a key step in applications such as medical diagnosis and chemical analysis [2]. In recent years, microfluidic technology has become an important means of particle focusing and separation, because of the advantages of small sample volume, high throughput, and simple control [3,4,5,6]. Among them, the viscoelastic microfluidics using viscoelastic fluids can achieve a single equilibrium position focusing of particles in a simple straight channel, and particles in viscoelastic fluids can also migrate to the walls or corners by overcoming the repulsion between the wall and the particles due to inertia. These interesting phenomena can be utilized and applied to particle focusing and separation through parameter control. A variety of experiments have been conducted for the further study of particle focusing and separation in viscoelastic fluids. The concept of elasto-inertial particle focusing was first proposed by Yang et al. [7], who realized the single-line focusing of particles in the center of the square channel by combining the elastic force and inertial lift. The two most important parameters for the migration of particles in the viscoelastic fluids are the Reynolds number *Re* (*Re* = *ρUL*/*η_t_*, *ρ* is the fluid density; *U* is the characteristic velocity; *L* = 2*hw*/(*h* + *w*) is the characteristic length, *h* and *w* are the height and width of channel, respectively; and *η_t_* is the total viscosity of the viscoelastic fluid) and Weissenberg number *Wi* (*Wi* = *λ_p_U*/*L*, *λ_p_* is the relaxation time of the polymer solution), and they represent the inertial and elastic effect, respectively. The ratio of the two parameters can be expressed as the elasticity number *El* = *Wi*/*Re*, which represents the relative importance of elasticity to the inertial effect, and the migration behavior of the particle is under the influence of *El* value. In addition, the bistable focusing of particles at the centerline and walls of the cylindrical channel can also be realized under strong shear thinning effect [8], and particle focusing can change from the original single-line focusing to multi-line focusing [9,10] or center plane focusing [11] in the various aspect ratios (AR) rectangular channels. For particle separation in viscoelastic fluids, particles can be usually characterized by size [12,13] and shape [14]. Size-based separation of particles is still the most widely employed method for separation experiments. Liu et al. [15] realized the separation of MCF-7 cells and RBCs mixture with AR = 1/2 microchannel, and *E.coli* bacteria and RBCs mixture in AR = 1/4 microchannel, respectively. In their research, small particles are focused at the center of the channel, while large particles are focused near the side walls of the channel. Nam et al. [16] realized the separation of white blood cells and candida cells in the channel with AR = 1/2, and then used the expansion channel to further enlarge the distance between the two kinds of cells.

Although the above reports show a wealth of particle migration characters and applications, the experiments have certain limitations in providing detailed information. The interaction between particles and fluid, the trajectories of particles on the cross-section of the channel, and the mechanism of particle migration are difficult to observe and analyze. However, these problems can be solved through numerical simulation which is helpful to further explain the mechanism of particle migration [17,18]. Raffiee et al. [19] used 3D numerical simulation to study the lift force distribution acting on particles in the viscoelastic fluid. They predicted the equilibrium positions under different parameters and analyzed the stability of the different equilibrium points. Yu et al. [20] used a fictitious domain method to study the focusing equilibrium positions of the particles in the square channel under different *Re* and *Wi*. In their study, as the fluid elasticity increases, the equilibrium positions occur successively at the cross-section midline, diagonal, corner, and the center of the channel. What is more, they also explored the possibility of the diagonal equilibrium positions in the channel with AR = 1/2. However, the reports on the three-dimensional numerical simulations of particle migration in viscoelastic fluids are still insufficient due to the complexity of viscoelastic fluids, the existence of fluid-particle interaction problems, and the demand for huge computing resources. In addition, most of previous research studies are numerical simulations of particle focusing in square channels. There are still relatively few studies on particle focusing in rectangular channels, especially in low-aspect-ratio channels and numerical simulations of different size particle separation in different aspect ratio channels with viscoelastic fluids.

In this paper, we employ the three-dimensional lattice Boltzmann method (LBM) coupled with the immersed boundary method (IBM) to simulate the migration of particles in the channels. LBM is a popular mesoscopic simulation program that can effectively deal with complex boundary geometries [21] and is suitable for parallel computing. The viscoelastic fluid is simulated by the LBM with two sets of distribution functions [22], one of them is used to calculate the fluid field evolution and the other is used to calculate the stress tensors. The interaction between particles and viscoelastic fluid is calculated by the IBM [23,24,25]. Through this method, particle focusing under different elastic numbers *El* is studied and the influence of cross-section AR on particle focusing is discussed. In addition, we also explored the size-based separation of particles in different cross-section AR channels, by studying the migration phenomenon of particles with different diameters.

## 2. Numerical Methods

### 2.1. 3D Lattice Boltzmann Method (LBM) of Viscoelastic Fluid

In the simulations, the dimensionless Navier–Stokes equations of incompressible flow are given by [26]:

(1) ∇·u=0,

(2) $$\rho [\frac{\partial u}{\partial t}+(u\cdot \nabla )u]=-\nabla p+\eta_{s}\nabla^{2}u+\nabla \cdot \tau +F_{D},$$

where *ρ*, ***u***, *p*, *η_s_*, ***τ***, and ***F***_D_ are the density, velocity, pressure, Newtonian solvent viscosity, viscoelastic stress tensor, and external force, respectively. Viscoelastic solvent can be regarded as a mixture of polymer and Newtonian solvent. The total viscosity of viscoelastic solvent *η*_t_ is the sum of polymer viscosity *η*_p_ and *η*_s_, and the parameter *β* = *η*_s_/*η*_t_ is used to describe the viscosity ratio. The external force ***F***_D_ is imposed to drive the flow. The Oldroyd-B constitutive equation [22] that describes viscoelastic fluids is given by:

(3) $$\lambda_{P}\frac{D\tau }{Dt}=\tilde{\nu }\nabla^{2}\tau +\lambda_{P}(\tau \cdot \nabla u+{\left(\nabla u\right)}^{T}\cdot \tau )-\tau +2\eta_{p}d,$$

where $\frac{D\tau }{Dt}=\frac{\partial \tau }{\partial t}+\left(u\cdot \nabla \right)\tau$ is the material derivative, *λ*_P_ is the polymer relaxation time, $\tilde{\nu }$ is the polymer diffusion parameter, $d=\left(\nabla u+{\left(\nabla u\right)}^{T}\right)/2$ is the rate of strains.

The viscoelastic flow field in microchannel is calculated by three-dimensional nineteen velocity (D3Q19) single relaxation time lattice Boltzmann Bhatnagar–Gross–Krook (LBGK) model [27], as follows:

(4) $$f_{\alpha }(x+c_{\alpha }\Delta t,t+\Delta t)=f_{\alpha }(x,t)+\frac{1}{\lambda }[f_{\alpha }^{eq}(\rho ,u)-f_{\alpha }(x,t)]+\Delta tF_{\alpha }(x,t),$$

where *α* is the 19 discrete directions in the D3Q19 model, *f_α_* (***x***, *t*) is the velocity distribution function at node ***x*** and time *t*, *λ* is the relaxation time, and ***c****_α_* is the discrete lattice velocity. The 19 discrete lattice velocity can be given by:

(5) $$c=c\left[\begin{array}{ccccccccccccccccccc} 0 & 1 & -1 & 0 & 0 & 0 & 0 & 1 & -1 & 1 & -1 & 1 & -1 & 1 & -1 & 0 & 0 & 0 & 0\\ 0 & 0 & 0 & 1 & -1 & 0 & 0 & 1 & -1 & -1 & 1 & 0 & 0 & 0 & 0 & 1 & -1 & 1 & -1\\ 0 & 0 & 0 & 0 & 0 & 1 & -1 & 0 & 0 & 0 & 0 & 1 & -1 & -1 & 1 & 1 & -1 & -1 & 1 \end{array}\right],$$

where *c*= Δ*x*/Δ*t*, Δ*x* and Δ*t* are the lattice spacing and the time step.

The $f_{\alpha }^{eq}(\rho ,u)$ in Equation (4) is equilibrium distribution function and can be calculated from the macroscopic fluid density *ρ* and velocity ***u*** as:

(6) $$f_{\alpha }^{eq}(\rho ,u)=\omega_{\alpha }\rho [1+3\frac{c_{\alpha }\cdot u}{c^{2}}+4.5\frac{{\left(c_{\alpha }\cdot u\right)}^{2}}{c^{4}}-1.5\frac{u^{2}}{c^{2}}],$$

where weight coefficients *ω*_0_=1/3, *ω*_1–6_=1/18, *ω*_7–18_=1/36.

The *F_α_* (***x***, *t*) in Equation (4) is external force term [28] and can be calculated as

(7) $$F_{\alpha }(x,t)=(1-\frac{1}{2\lambda })\omega_{\alpha }[3\frac{c_{\alpha }-u}{c^{2}}+9\frac{c_{\alpha }\cdot u}{c^{4}}c_{\alpha }]\cdot F,$$

where ***F*** = ***F***_D_ + ***F***_A_ + ***F***_V_ is the total external force acting on the fluid. ***F***_D_, ***F***_A_, and ***F***_V_ are the driving force, the acting force from particles, and the elastic force, respectively.

The macroscopic fluid density *ρ*, velocity ***u***, and kinematic viscosity *υ* of the fluid can be given as:

(8) $$\rho =\sum_{\alpha }f_{\alpha },\;\rho u=\sum_{\alpha }c_{\alpha }f_{\alpha }+\frac{1}{2}F\Delta t,\;\upsilon =\frac{1}{3}\frac{\Delta x^{2}}{\Delta t}(\lambda -\frac{1}{2}).$$

The elastic force $F_{V}=\nabla \cdot \tau$ is the calculation result from the stress tensor ***τ*** of the viscoelastic flow. The stress tensor ***τ*** can be computed from the tensor distribution function *G_ijα_* (***x***, *t*) [22] which is on the same lattice node of *f_α_* (***x***, *t*). The LBGK model of *G_ijα_* (***x***, *t*) is also based on D3Q19 as:

(9) $$G_{ij\alpha }(x+{\tilde{c}}_{\alpha }\Delta \tilde{t},t+\Delta \tilde{t})=G_{ij\alpha }(x,t)+\frac{1}{\tilde{\lambda }}[G_{ij\alpha }^{eq}(x,t)-G_{ij\alpha }(x,t)]+\Delta \tilde{t}\chi_{ij\alpha }(x,t)+0.5{\left(\Delta \tilde{t}\right)}^{2}\partial_{t}\chi_{ij\alpha }(x,t),$$

where *G_ijα_* (***x***, *t*) is the distribution function at node ***x*** and time *t*, $\Delta \tilde{t}$, and ${\tilde{c}}_{\alpha }$ are the time step and discrete lattice velocity of *G_ijα_* (***x***, *t*), $\tilde{\lambda }$ is the relaxation parameter, $G_{ij\alpha }^{eq}(x,t)$ is the equilibrium tensor distribution function and can be calculated as:

(10) $$G_{ij\alpha }^{eq}(x,t)=\omega_{\alpha }\tau_{ij}\left[1+3\frac{{\tilde{c}}_{\alpha }\cdot u}{{\tilde{c}}^{2}}+4.5{\frac{\left({\tilde{c}}_{\alpha }\cdot u\right)}{{\tilde{c}}^{4}}}^{2}-1.5\frac{u^{2}}{{\tilde{c}}^{2}}\right],$$

where $\tilde{c}=\Delta x/\Delta \tilde{t}$, *τ_ij_* is the component of ***τ***.

The source term *χ_ijα_* in Equation (9) can be given as:

(11) $$\chi_{ij\alpha }=\omega_{\alpha }\chi_{ij}[1+3(\frac{\tilde{\lambda }-1/2}{\tilde{\lambda }})\frac{{\tilde{c}}_{\alpha }\cdot u}{{\tilde{c}}^{2}}],$$

where *χ_ij_* is the component of tensor ***χ*** which can be given as:

(12) $$\chi =\tau \cdot \nabla u+{\left(\nabla u\right)}^{T}\cdot \tau +\frac{1}{\lambda_{p}}(2(1-\beta )\eta_{t}d-\tau ).$$

The stress tensor ***τ*** of the viscoelastic flow can be obtained through:

(13) $$\tau_{ij}=\sum_{\alpha }G_{ij\alpha }(x,t)=\sum_{\alpha }G_{ij\alpha }^{eq}(x,t),\tau =\left[\begin{array}{ccc} \tau_{11} & \tau_{12} & \tau_{13}\\ \tau_{21} & \tau_{22} & \tau_{23}\\ \tau_{31} & \tau_{32} & \tau_{33} \end{array}\right],$$

and then the elastic force ***F***_V_ can be calculated.

In this study, periodic boundary condition is applied to simulate an infinite length channel and non-slip boundary condition is applied for walls. The distribution function *f_α_* (***x***, *t*) and *G_ijα_* (***x***, *t*) on the walls are calculated by non-equilibrium extrapolation method [29], as follows:

(14) $$f_{\alpha }(x,t)=f_{\alpha }^{eq}(\rho_{f},u_{w})+[f_{\alpha }(x_{f},t)-f_{\alpha }^{eq}(x_{f},t)],$$

(15) $$G_{ij\alpha }(x,t)=G_{ij\alpha }^{eq}(\tau_{ijf},u_{w})+[G_{ij\alpha }(x_{f},t)-G_{ij\alpha }^{eq}(x_{f},t)],$$

where ***u****_w_* = 0 is the velocity of walls, *ρ_f_* and *τ_ijf_* are the density and stress tensor component of adjacent fluid lattice. $f_{\alpha }(x_{f},t)$, $G_{ij\alpha }(x_{f},t)$ and $f_{\alpha }^{eq}(x_{f},t)$, $G_{ij\alpha }^{eq}(x_{f},t)$ are the distribution functions and equilibrium distribution functions of adjacent fluid nodes.

### 2.2. IBM for the Interaction between Particles and Fluid

In the simulations, particle can be built through an 3D finite element membrane model which divides the surface of the particle into *N* triangular surface elements. The vertices of all triangular elements constitute the nodes of the particle surface boundary. The information of particle surface nodes and fluid nodes can be transferred to each other through IBM [30] to realize the interaction between particles and fluid. The total energy *E* of the particle deformation produced by fluid shear is calculated from *E* = *E*_S_ + *E*_B_ + *E*_A_ + *E*_V_, where *E*_S_, *E*_B_, *E*_A_, and *E*_V_ denote strain energy, bending energy, area energy, and volume energy, respectively, and can be calculated as:

(16) $$E_{S}=\sum_{n=1}^{N}E_{\mathrm{ST}n},E_{\mathrm{ST}}=\frac{\kappa_{S}}{12}(I_{1}^{2}+2I_{1}-2I_{2})+\frac{\kappa_{\alpha }}{12}I_{2}^{2},$$

(17) $$E_{B}=\frac{\kappa_{B}}{2}\sum_{n=1}^{\frac{3N}{2}}{\left(\phi_{n}-\phi_{n,0}\right)}^{2},$$

(18) $$E_{A}=\frac{\kappa_{A}}{2}\frac{{\left(A-A_{0}\right)}^{2}}{A_{0}},$$

(19) $$E_{V}=\frac{\kappa_{V}}{2}\frac{{\left(V-V_{0}\right)}^{2}}{V_{0}},$$

where $I_{1}=\lambda_{1}^{2}+\lambda_{2}^{2}-2$ and $I_{2}=\lambda_{1}^{2}\lambda_{2}^{2}-1$ are strain invariants (*λ*_1_, *λ*_2_ are eigenvalues). $\kappa_{S}$, $\kappa_{\alpha }$, $\kappa_{B}$, $\kappa_{A}$, and $\kappa_{V}$ are the surface elastic shear modulus, area dilation modulus, binding modulus, surface modulus, and volume modulus, respectively. When the elastic moduli are large enough, the simulated particle can be regarded as a rigid particle. $\phi_{n}$ and $\phi_{n,0}$, *A* and *A*_0_, *V* and *V*_0_ are the angle between normal vectors of adjacent triangular faces, particle surface area, particle volume after, and before the particle deformation, respectively.

Then the restoring force on particle nodes can be calculated from $f(x_{n})=-\partial E/\partial x_{n}$, where ***x****_n_* is the coordinate of the particle node. Similarly, the coordinate of the fluid node is expressed by ***x****_f_*. The acting force ***F***_A_ from particle on ***x****_f_* can be calculated by IBM as:

(20) $$F_{A}(x_{f})=\sum {}_{p}f(x_{n})D(x_{n}-x_{f}),$$

where $D(x_{n}-x_{f})=\delta (x_{n}-x_{f})(y_{n}-y_{f})(z_{n}-z_{f})$ and:

(21) $$\delta (r)=\{\begin{array}{cc} \frac{1}{4}(1+\cos(\frac{\pi r}{2})), & \left|r\right|\leq 2\\ 0, & \left|r\right|>2 \end{array}.$$

At this time, ***F***_D_, ***F***_A_, and ***F***_V_ in the force term ***F*** have all been obtained. The velocity distribution $u_{f}\left(x_{f}\right)$ of the flow field can be obtained by calculating Equations (4)–(8). The velocity distribution $u_{p}\left(x_{n}\right)$ of the particle node can be updated by $u_{p}(x_{n})=\sum_{f}u_{f}(x_{f})D(x_{f}-x_{n})$, which completes the interaction between particle and fluid for one time step.

### 2.3. Channel Model and Validation

**Figure 1:**
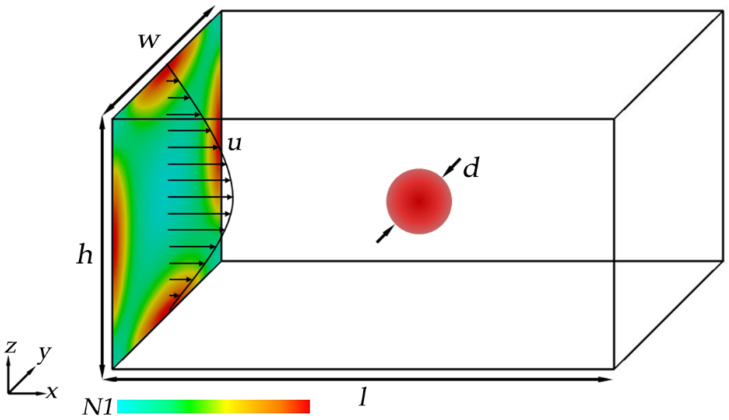
Schematic diagram of the 3D channel model.

The schematic diagram of the particles in the straight channel with viscoelastic fluid is shown in Figure 1. For a rectangular channel, the height and width are *h* = 40 μm, *w* = *h*/AR, and for the square channel, *w* = *h* = 50 μm. The trajectories of particles in the viscoelastic fluid can be simulated by the method given above. In the simulation, the fluid can be driven by driving force *F*_D_. Thus, the acceleration *a* of the fluid can be obtained from the Poiseuille equation as *a* = 32*υ*^2^*Re/L*^3^. The channel can be simulated with infinite length in the *x*-axis direction benefiting from the periodic boundary conditions of the inlet and outlet. The number of membranes *N* of 6 μm and 12 μm diameter particles in the simulation are 120 and 480, respectively. The elastic moduli for a rigid particle are set as $\kappa_{S}$ = 3.2 × 10^−1^ N/m, $\kappa_{\alpha }$ = 3.2 × 10^−1^ N/m, $\kappa_{B}$ = 3.2 × 10^−13^ Nm, $\kappa_{A}$ = 3.2 × 10^−2^ N/m, and $\kappa_{V}$ = 3.2 × 10^4^ N/m^2^. The other parameters in simulation can be given as Δ*x* = 1.0 × 10^−6^ m, Δ*t* = $\Delta \tilde{t}$ = 1.0 × 10^−7^ s, *υ* = 1.0 × 10^−6^ m^2^/s, *β* = 0.3.

**Figure 2:**
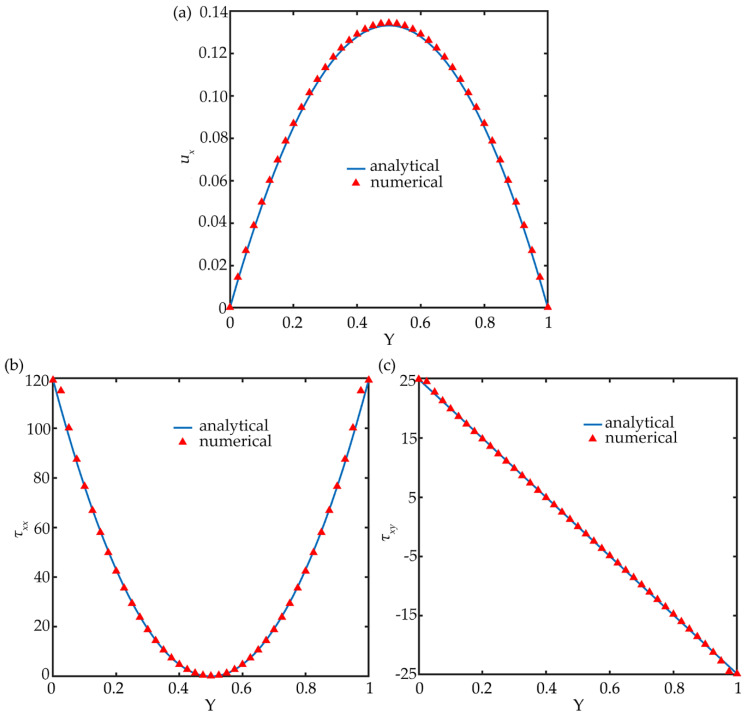
Numerical and analytical results of the velocity distribution (**a**), the normal-stress components *τ_xx_* (**b**), and the extra-stress components *τ_xy_* (**c**) at the channel center plane under *Wi* = 0.3, *Re* = 1.0, and *β* = 0.3.

To validate the numerical simulation model, the particle-free viscoelastic fluid evolution and the particle rotation in viscoelastic shear fluid are conducted respectively. For the particle-free fluid model, a microtube with a circle cross-section is employed and the simulation parameters are set as *Re* = 1.0, *Wi*= 0.3, and *β* = 0.3. The numerical and analytical results of the velocity distribution, normal-stress components *τ_xx_*, and extra-stress components *τ_xy_* at the channel center plane are shown in Figure 2. The simulation results are in good agreement with the analytical solutions. The components of stress tensor *τ* can be calculated as:

(22) $$\tau_{xx}=2\lambda_{p}\eta_{t}(1-\beta ){\left(\frac{\partial u}{\partial y}\right)}^{2},$$

(23) $$\tau_{xy}=\eta_{t}(1-\beta )(\frac{\partial u}{\partial y}).$$

**Figure 3:**
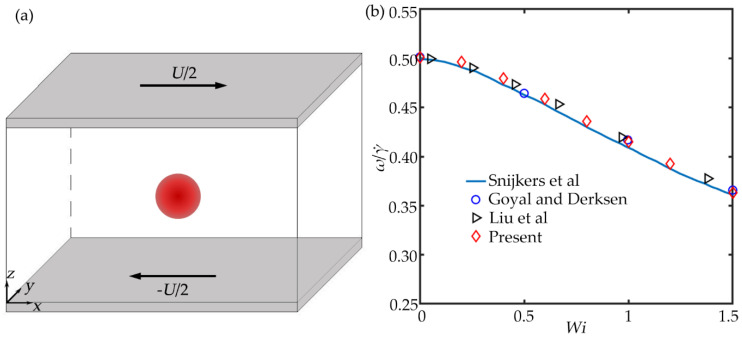
(**a**) Schematic diagram of particle in an Oldroyd-B shear flow. (**b**) The angular velocity of a single spherical particle in the Oldroyd-B shear flow under different *Wi* compared with the results of Snijkers et al. [31], Goyal et al. [32], and Liu et al. [33].

For fluid–particle interaction, the simulation of a spherical particle rotation in an Oldroyd-B viscoelastic shear flow is employed to validate the present method. The shear flow model is shown in Figure 3a. In the shear flow model, the velocities on the upper and lower board nodes are set as the same constant values with opposite directions. Periodic boundary conditions are applied around the fluid field, and the particle is placed at the fluid filed center. The angular velocities of the particle are calculated under different *Wi*, as shown in Figure 3b. The angular velocity of particle decreases monotonously as *Wi* increases, and the present results agree well with the previous results.

## 3. Results and Discussion

**Figure 4:**
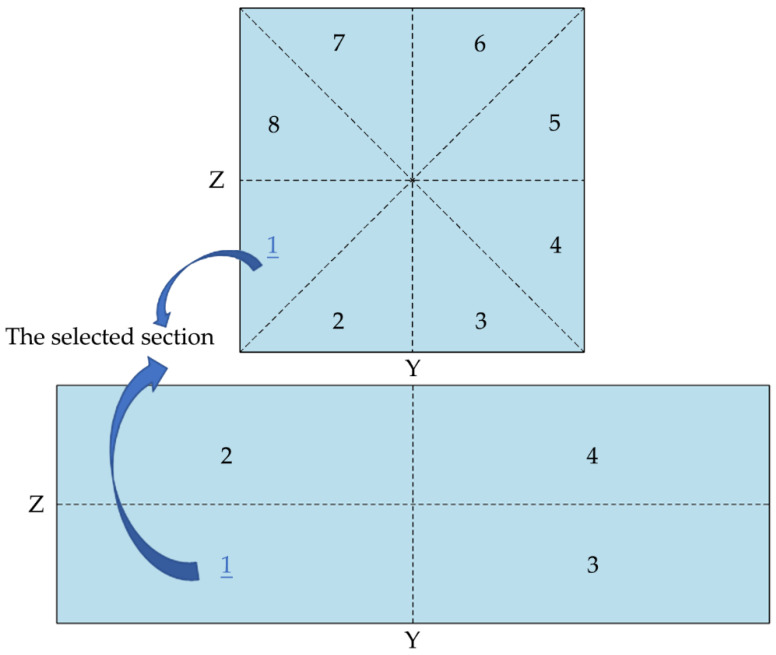
Schematic diagram of the selected simulated section on the cross-section.

The shape of cross-section, velocity distribution, and stress tensor distribution on the cross-section are all symmetrical. Therefore, the square cross-section can be divided into 8 sections according to its four symmetry axes (the rectangular cross-section is divided into 4 sections), as shown in Figure 4. The trajectories of the particle on the entire cross-section can be obtained by only simulating the migration of particle on Section 1.

### 3.1. Particle Focusing

**Figure 5:**
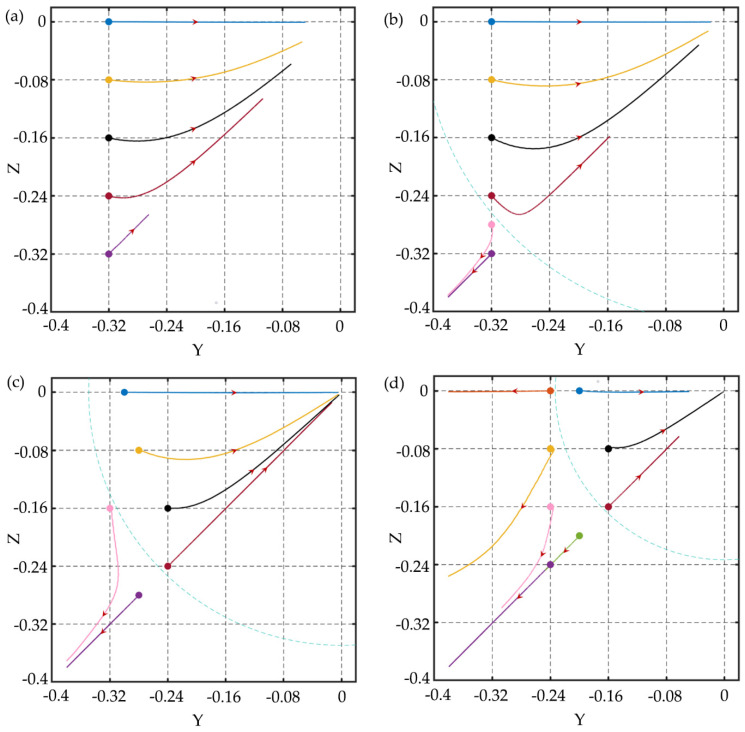
The migration trajectories of particles at different initial positions of the cross-section in the square channel under *Re* = 1.0, (**a**) *Wi* = 0.05, *El* = 0.05; (**b**) *Wi* = 0.1, *El* = 0.1; (**c**) *Wi* = 0.3, *El* = 0.3; (**d**) *Wi* = 0.6, *El* = 0.6. In (**b**–**d**), the blue dotted lines are the separatrices. The maximum simulation time in the figures is 1,200,000 dimensionless time units.

In the square channel, the particle with a diameter of 6 μm (*d*/*h* = 0.12) is released at different initial positions on Section 1 under different elasticity numbers. The Reynolds number is *Re* = 1.0 and the Weissenberg number increases from 0.05 to 0.6, which makes the *El* range from 0.05 to 0.6. The migration trajectories of particle at different initial positions of the cross-section are shown in Figure 5. In the particle trajectory figures, the dots denote the initial positions of the particles, the lines of different colors correspond to the migration trajectories of the particles at different initial positions of the cross-section, and the arrow is the moving direction of the particle on the cross-section. For *El* = 0.05, particles at different initial positions all migrate towards the centerline of the channel and the particles migrate slowly due to the small elastic force. As *El* increases to 0.1, the efficiency of particle focusing increases, and the particle close to the corner migrates towards the corner instead of the centerline of the channel. What is more, there seems to be a separatrix in the channel, the particle within the separatrix migrates towards the channel centerline and the particle outside the separatrix migrates towards the wall or corner. This phenomenon is consistent with previous experimental [8,34,35] and numerical [20,36] results. For *El* = 0.3, the separatrix shrinks, and the particles close to the wall migrate towards the corner. As *El* further increases to 0.6, this phenomenon becomes more obvious, and the separatrix shrinks further. These phenomena indicate that there always has a centerline focusing equilibrium position under different *El*. For small *El*, the particle focusing efficiency is slow, but for large *El*, some particles migrate to corners and single-line focusing cannot be achieved. Therefore, *El* is the key parameter to obtain different focusing patterns. In addition, the trajectories of the particles at the symmetry axes are relatively straight due to the symmetry of the fluid field, while the particles on the other positions deflect towards the diagonal during the migration process. Yu et al. [20] also discovered this phenomenon, and they realized the particle focusing on the diagonal by increasing *Re*. In their research, the diagonal equilibrium position exists under the conditions of (*Re* = 10, *Wi* = 0.05, *El* = 0.005), (*Re* = 50, *Wi* = 0.25, *El*= 0.005), and (*Re*= 100, *Wi* = 0.5, *El* = 0.005), but as *El* increases, the particles will migrate to the center of the channel.

**Figure 6:**
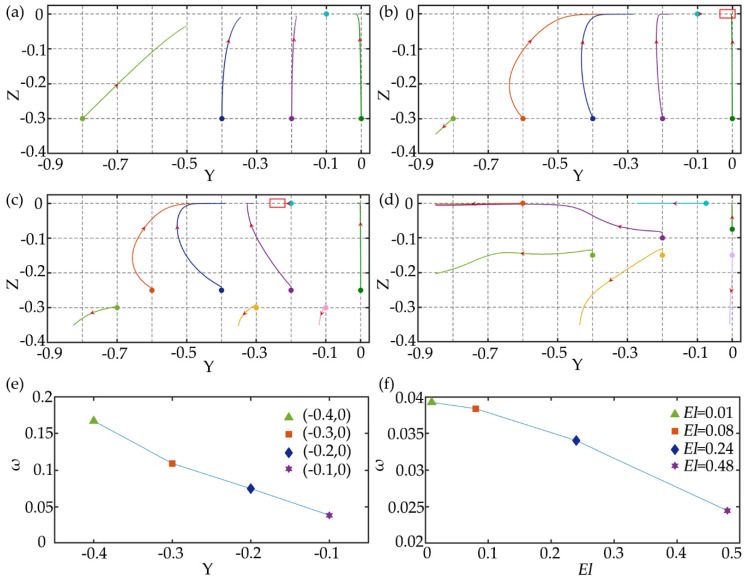
The migration trajectories of particles with the diameter of 6 μm (*d*/*h* = 0.15) at different initial positions of the cross-section in channel with AR =1/2 under *Re* = 2.5, (**a**) *Wi* = 0.005, *El* = 0.01; (**b**) *Wi* = 0.2, *El* = 0.08; (**c**) *Wi* = 0.6, *El* = 0.24; (**d**) *Wi* = 1.2, *El* = 0.48. The red boxes in the figures are the predicted particle focusing equilibrium positions. The maximum simulation time in the figures is 1,200,000 dimensionless time units. (**e**) Particle rotation speed at (−0.4, 0), (−0.3, 0), (−0.2, 0), and (−0.1, 0) under *Wi* = 0.2, *Re* = 2.5, *El* = 0.08. (**f**) Particle rotation speed at (−0.1, 0) under different *El*.

For the rectangular channel, the channel with AR = 1/2 is studied firstly. Similarly, the performance of particle focusing under different *El* is compared. The Reynolds number is *Re* = 2.5 and the Weissenberg number increases from 0.025 to 1.2, which makes the *El* range from 0.01 to 0.48. The migration trajectories of particle at different initial positions of the cross-section are shown in Figure 6a–d. For *El* = 0.01, the particles migrate very slowly, and the elastic forces acting on the particles are very small and not enough to make the particles fully focused to the channel center. For *El* = 0.08, the efficiency of particle focusing increases, and the particles close to the corner migrate towards the corners, which is consistent with the square channel. However, the longitudinal migrations of particles are significantly faster than the lateral migrations, and the particles are firstly focused at the long midline plane (z = 0) and then migrate towards the equilibrium position at the centerline. For the particles at the long midline plane, due to the smaller inertial lift and elastic force, particles close to the channel center migrate more slowly. Meanwhile, particles close to the channel center also have slower rotation speed as shown in Figure 6e. As *El* increases to 0.24, particle focusing becomes more interesting, the off-centerline focusing equilibrium position appears, which means that the particles can be focused into double lines or even multiple lines. Compared with *El* = 0.08, since the large *El* may attenuate the particle rotation, and the particle rotation speed is further reduced as shown in Figure 6f, which weakens the fluid stretching [37] and makes the elastic force directing to the channel center smaller, so that the particle can be focused at the off-centerline equilibrium position. As *Wi* further increases to 0.48, the equilibrium positions of the particles change again, almost all the particles migrate towards the walls and corners.

**Figure 7:**
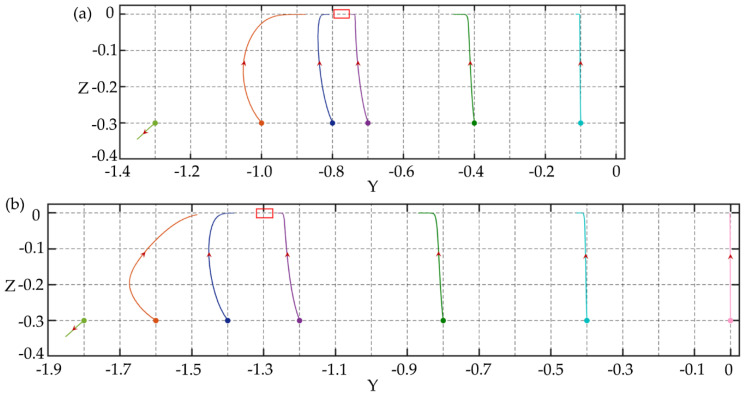
The migration trajectories of particles with *d*/*h*=0.15 in channels with different aspect ratios: (**a**) AR = 1/3 under *Re* = 2.5 and *Wi* = 0.2; (**b**) AR = 1/4 under *Re* = 2.5 and *Wi* = 0.2. The red boxes in the figures are the predicted particle focusing equilibrium positions. The maximum simulation time in the figures is 1,200,000 dimensionless time units.

Channels with lower aspect ratios are also studied. Figure 7 shows the migration trajectories of particles with the diameter of 6 μm (*d*/*h*=0.15) in the channels with the aspect ratio of 1/3 and 1/4 under *Re* = 2.5 and *Wi* = 0.2. Compared with Figure 6b, with the aspect ratio decreasing, the focusing equilibrium position is farther away from the centerline, and the lateral migration speed of particles at the long midline plane further slows down. Within the same time (1,200,000 dimensionless time units), particles in the channel with the aspect ratio of 1/2 can be focused near the equilibrium position, while particles in the channel with the aspect ratio of 1/4 only migrate to the long midline plane. We can predict that when the aspect ratio is further reduced, the phenomenon that particles are focused at the entire long midline plane will be formed. Seo et al. [11] confirmed this prediction and realized the focusing of the long midline plane of the red blood cells (RBCs) by using a low aspect ratio channel with the height of 50 μm and the width of 500 μm, and used a holographic microscope to perform quantitative phase imaging on the midline plane to achieve the monitoring and counting of RBCs. This plane focusing can make the height of particles is highly uniform, which reduces the out-of-focus blurring and improves the detection sensitivity. In addition, for particles whose initial positions are close to the center of the channel, it is difficult to migrate laterally after they migrate to the long midline plane. Therefore, particles are actually focused at the region between the two equilibrium positions on the long midline plane, and the smaller the aspect ratio, the larger the region is.

**Figure 8:**
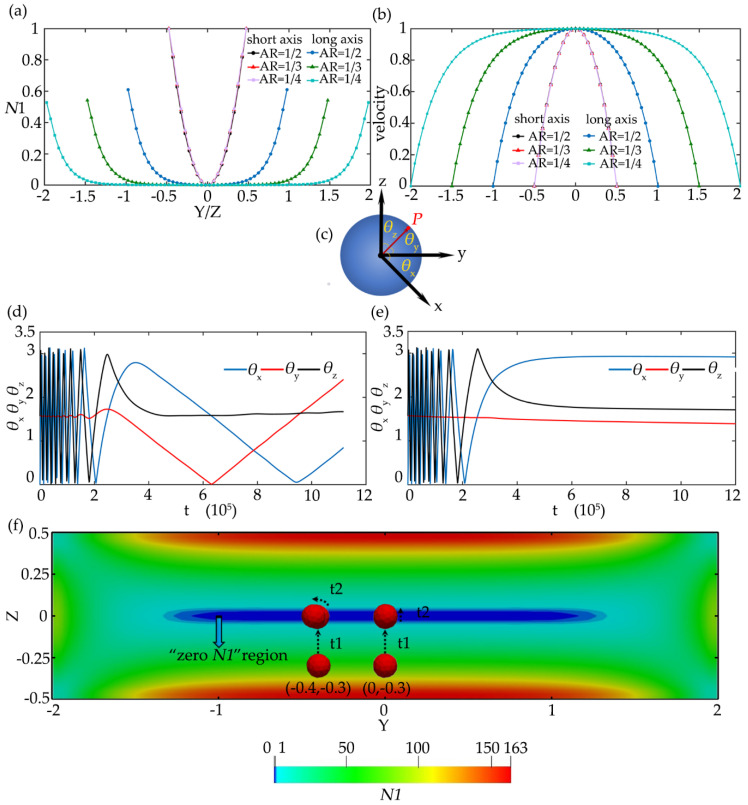
The non-dimensionalized the first normal stress difference *N*_1_ distributions (**a**) and velocity distribution (**b**) on the long symmetry axis and the short symmetry axis of the cross section of the channel with AR = 1/2, 1/3, and 1/4. (**c**) Schematic diagram of the vector ***P*** and the angles *θ_x_*, *θ_y_*, and *θ_z_* between the vector ***P*** and the coordinate axis. Time history of *θ_x_*, *θ_y_*, and *θ_z_* of the particle with *d*/*h* = 0.15 in the channel with AR = 1/4 at the initial position (**d**) (−0.4, −0.3) and (**e**) (0, −0.3). (**f**) Schematic diagram of particle migration on the cross-section of the channel, *t*1 = 300,000 dimensionless time units, *t*2 = 900,000 dimensionless time units.

To further explore the particle migration in the channels with AR < 1, the stress distribution and particle rotation on the cross-section are studied. In the rectangular channel, the velocity distribution and stress distribution on the long and short symmetry axis of the cross-section are different. The first normal stress difference *N*_1_ on the long symmetry axis is significantly smaller than that on the short symmetry axis, as shown in Figure 8a, and there is a larger “zero *N*_1_” region (*N*_1_ is close to zero in this region) along the long symmetry axis, and this region increases as the aspect ratio decreases. Since the elastic force acting on the particles is proportional to *N*_1_ [38], the elastic force acting on the particle in the “zero *N*_1_” region is small and close to zero. Moreover, for particles near the center on the long symmetry axis, especially for AR = 1/4, the velocity difference around the particles is also very small, as shown in Figure 8b. It means that the shear-induced inertial lift and wall-induced inertial lift (the particles are far from the wall) acting on the particles are very small.

For particle rotation, the rotation process of the particle is described by three angles *θ_x_*, *θ_y_*, and *θ_z_*, which are the angles between the vector ***P*** and the *x*-axis, *y*-axis, and *z*-axis, respectively, as shown in Figure 8c. The vector ***P*** points from the particle center to a fixed node on the surface of the particle. The initial vector ***P*** is set along the *x*-axis, and the changes of *θ_x_*, *θ_y_*, and *θ_z_* with particle migration are shown in Figure 8d,e. The *θ_y_* remains unchanged at the beginning, which indicates that the particle rotates around the y-axis. After the particle is focused at the long midline plane, for (0, −0.3), the particle hardly rotates, and for (−0.4, −0.3), the particle turns to rotate around the z-axis, but the rotation speed is very slow. This proves that the velocity shear around particles is small. Figure 8f more clearly shows the migration trajectories of the two particles on (0.4, −0.3) and (0, −0.3). The particles migrate to the long midline plane after the time *t*1 (*t*1 = 300,000 dimensionless time units), but hardly migrate laterally during the longer time *t*2 (*t*2 = 900,000 dimensionless time units). Therefore, in the “zero *N*_1_” region, both the elastic and inertial effects acting on particles are small. The particles are relatively stable and difficult to migrate laterally. The above results are obtained under *Re* = 2.5, *Wi* = 0.2, *El* = 0.08.

### 3.2. Particle Separation

In the viscoelastic fluid of straight microchannel, equilibrium position of particles may differ with various diameters, which can be utilized to particle separation [15,16]. To study the different size particle performance in various AR microchannels, the trajectories of particle with diameters of 6 and 12 μm are calculated and systematically analyzed.

**Figure 9:**
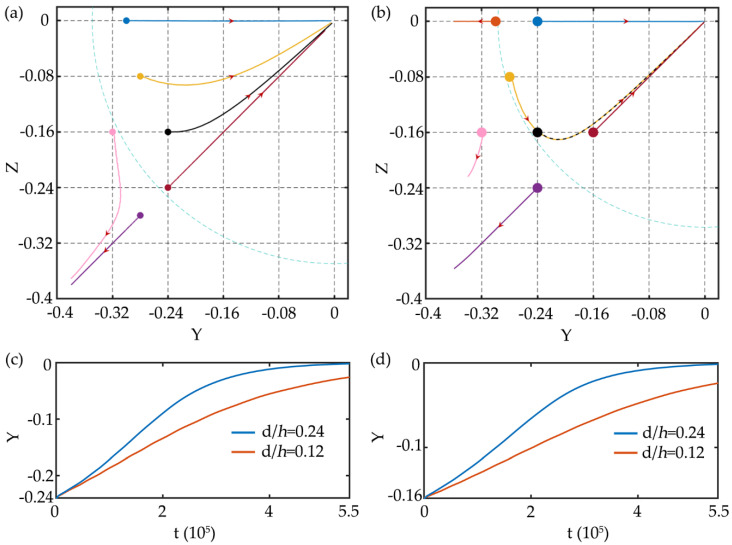
The migration trajectories of the particles with (**a**) *d*/*h* = 0.12 and (**b**) *d*/*h* = 0.24 at different initial positions of the cross-section under *Re* = 1.0 and *Wi* = 0.3. The maximum simulation time for (**a**) and (**b**) is 1,200,000 dimensionless time units. Time history of the longitudinal migration of particles at the initial positions (**c**) (−0.24, 0) and (**d**) (−0.16, −0.16).

In the square channel, the migration trajectories of particles with diameters of 6 and 12 μm (*d*/*h* = 0.12 and 0.24) under *Re* = 1.0 and *Wi* = 0.3 are shown in Figure 9a,b. The small particle has a larger region within the separatrix and more of them can be focused to the centerline of the channel. The large particle has a faster lateral migration speed as shown in Figure 9c,d. It is difficult to realize particle separation in the square channel due to the same focusing equilibrium positions (the center and corner of the channel) of the large and small particles. Even if the initial position of the mixed particles can be set near the wall and corner with the help of sheath flow, so that some small particles can migrate to the center of the channel while large particles migrate to the corner, the separation efficiency is extremely low. It is why few researchers use square channel for particle separation. However, the characteristic that large particles have a faster lateral migration speed to the centerline can be utilized in the sudden expansion channel. Nam et al. [39,40] proposed a two-stage flow channel structure for particle separation. In the first stage, particles with different sizes are focused to the center of the channel, and then in the second stage, large particles can be separated from small particles in the sudden expansion channel due to the faster migration. In addition, large particles are easier to deflect towards diagonal line during the migration, which may be caused by the larger velocity shear around the large particles.

**Figure 10:**
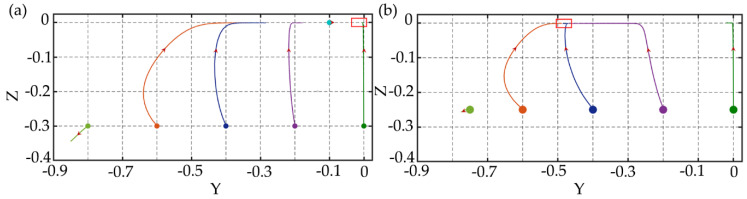
The migration trajectories of particles with different diameters in the channel with aspect ratio of AR = 1/2 under *Re* = 2.5 and *Wi* = 0.2, (**a**) *d*/*h* = 0.15; (**b**) *d*/*h* = 0.30. The red boxes in the figures are the predicted particle focusing equilibrium positions. The maximum simulation time in the figures is 1,200,000 dimensionless time units.

**Figure 11:**
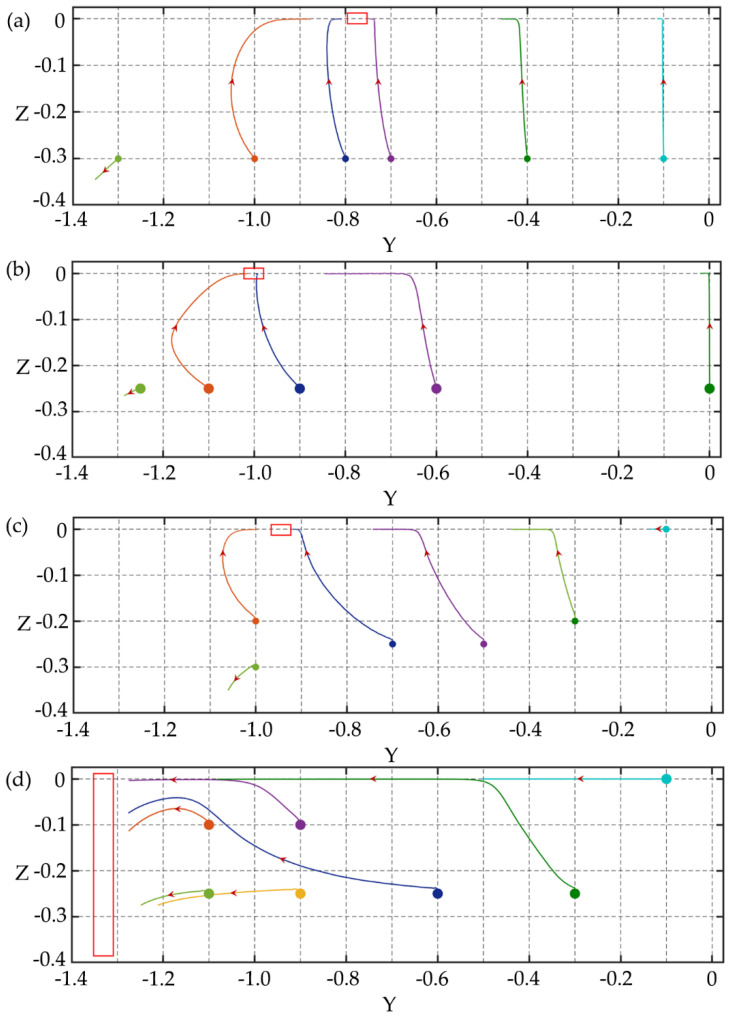
The migration trajectories of particles with different diameters in the channel with aspect ratio of AR = 1/3: (**a**) *d*/*h* = 0.15 under *Re* = 2.5 and *Wi* = 0.2; (**b**) *d*/*h* = 0.30 under *Re* = 2.5 and *Wi* = 0.2; (**c**) *d*/*h* = 0.15 under *Re* = 2.5 and *Wi* = 0.6; (**d**) *d*/*h* = 0.30 under *Re* = 2.5 and *Wi* = 0.6. The red boxes in the figures are the predicted particle focusing equilibrium positions. The maximum simulation time in the figures is 1,200,000 dimensionless time units.

In the rectangular channel, particles of various diameters have different focusing equilibrium positions for separation and the problems in the square channel disappear. Figure 10 shows the migration trajectories of particles with *d*/*h* = 0.15 and 0.30 in the channel with the aspect ratio of 1/2 under *Re* = 2.5, *Wi* = 0.2, and *El* = 0.08. The focusing equilibrium position of small particles is at the center of the channel, while large particles migrate towards off-center equilibrium positions. This is consistent with the results of Liu et al. [15] and Nam et al. [16]. The channel with smaller aspect ratios is also studied. Figure 11 shows the migration trajectories of particles with the diameters of *d*/*h* = 0.15 and 0.30 in the channel with the aspect ratio of 1/3 under different *El*. For *El* = 0.08, the migration performance of large particles is similar to small ones, but the focusing equilibrium position of large particles is farther away from the centerline of the channel than small particles. As *El* increases to 0.24, the elastic effect acting on the particles is enhanced. For small particles, the equilibrium position is farther away from the centerline of the channel, while for large particles, almost all of them migrate towards the walls and corners with faster lateral migration speed. Comparing the equilibrium positions of different size particles in *El* = 0.08 and 0.24 rectangular channels, *El* has a greater influence on the migration of large particles. When *El* = 0.24, the large particle can be fully focused near the channel walls and the separation efficiency for different size particles is higher than that of the *El* = 0.08 channel.

Overall, rectangular channel is a better design for particle separation than the square channel. What is more, the 1/2 AR rectangular channel can realize center focusing for small particles, while the 1/3 AR channel is able to focus the large particles fully near the sidewall. In addition, both of them can be applied to particle separation device according to the specific requirement.

## 4. Conclusions

In summary, we systematically explore the elasto-inertial focusing and separation of particles in viscoelastic fluids with different aspect ratios through the numerical simulation. For particle focusing, a single line focusing at the channel center can be achieved in a square channel. In the rectangular channel, particle focusing becomes more complicated and interesting. With the aspect ratio decreasing, the double-line focusing, multi-line focusing, and even plane focusing of particles will appear. The equilibrium position of particle also can be controlled by parameter *El*. More particles can be focused at the corners under a larger *El* in the square channel, while in the rectangular channel, the equilibrium positions of particles can be moved closer to the side walls with *El* increasing.

For particle separation, large particles have a faster lateral migration speed and a smaller region within the separatrix, but cannot be effectively separated from the small particles in the square channel, because of their same focusing equilibrium position at the centerline. In the rectangular channel, large and small particles have different focusing equilibrium positions, and the large particles with equilibrium positions closer to the wall can be separated from small ones. Moreover, *El* has a greater influence on the migration of large particles, and higher *El* can help large particles to be focused closer to the wall, while small particles stay near the center of the channel at the long midline plane. We expect the results will be helpful for the design of microfluidic chips for particle/cell focusing and separation.

## References

[1] Xuan X., Zhu J., Church C. (2010). Particle focusing in microfluidic devices. Microfluid. Nanofluid..

[2] Lim H., Nam J., Shin S. (2014). Lateral migration of particles suspended in viscoelastic fluids in a microchannel flow. Microfluid. Nanofluid..

[3] Amini H., Lee W., Carlo D.D. (2014). Inertial microfluidic physics. Lab. Chip.

[4] Yan S., Tan S.H., Li Y., Tang S., Teo A.J.T., Zhang J., Zhao Q., Yuan D., Sluyter R., Nguyen N.T. (2018). A portable, hand-powered microfluidic device for sorting of biological particles. Microfluid. Nanofluid..

[5] Di Carlo D. (2009). Inertial microfluidics. Lab. Chip.

[6] Tang W., Jiang D., Li Z., Zhu L., Shi J., Yang J., Xiang N. (2019). Recent advances in microfluidic cell sorting techniques based on both physical and biochemical principles. Electrophoresis.

[7] Seungyoung Y., Jae Young K., Seong Jae L., Sung Sik L., Min K.J. (2011). Sheathless elasto-inertial particle focusing and continuous separation in a straight rectangular microchannel. Lab. Chip.

[8] D’Avino G., Romeo G., Villone M.M., Greco F., Netti P.A., Maffettone P.L. (2012). Single line particle focusing induced by viscoelasticity of the suspending liquid: Theory, experiments and simulations to design a micropipe flow-focuser. Lab. Chip.

[9] Xiang N., Dai Q., Ni Z. (2016). Multi-train elasto-inertial particle focusing in straight microfluidic channels. Appl. Phys. Lett..

[10] Yang S.H., Lee D.J., Youn J.R., Song Y.S. (2017). Multiple-line particle focusing under viscoelastic flow in a microfluidic device. Anal. Chem..

[11] Seo K.W., Ha Y.R., Lee S.J. (2014). Vertical focusing and cell ordering in a microchannel via viscoelasticity: Applications for cell monitoring using a digital holographic microscopy. Appl. Phys. Lett..

[12] Liu C., Guo J., Tian F., Yang N., Yan F., Ding Y., Wei J., Hu G., Nie G., Sun J. (2017). Field-free isolation of exosomes from extracellular vesicles by microfluidic viscoelastic flows. ACS Nano.

[13] Zhou Y., Ma Z., Tayebi M., Ai Y. (2019). Submicron particle focusing and exosome sorting by wavy microchannel structures within viscoelastic fluids. Anal. Chem..

[14] Lu X., Xuan X. (2015). Elasto-inertial pinched flow fractionation for continuous shape-based particle separation. Anal. Chem..

[15] Liu C., Xue C., Chen X., Shan L., Tian Y., Hu G. (2015). Size-Based separation of particles and cells utilizing viscoelastic effects in straight microchannels. Anal. Chem..

[16] Nam J., Jang W.S., Hong D.H., Lim C.S. (2019). Viscoelastic separation and concentration of fungi from blood for highly sensitive molecular diagnostics. Sci. Rep. UK.

[17] Villone M.M., D’Avino G., Hulsen M.A., Greco F., Maffettone P.L. (2011). Simulations of viscoelasticity-induced focusing of particles in pressure-driven micro-slit flow. J. Non-Newton Fluid.

[18] Villone M.M., D’Avino G., Hulsen M.A., Greco F., Maffettone P.L. (2013). Particle motion in square channel flow of a viscoelastic liquid: Migration vs. secondary flows. J. Non-Newton Fluid.

[19] Raffiee A.H., Ardekani A.M., Dabiri S. (2019). Numerical investigation of elasto-inertial particle focusing patterns in viscoelastic microfluidic devices. J. Non-Newton Fluid.

[20] Yu Z., Wang P., Lin J., Hu H.H. (2019). Equilibrium positions of the elasto-inertial particle migration in rectangular channel flow of Oldroyd-B viscoelastic fluids. J. Fluid Mech..

[21] Chen Q., Zhang X.B., Li Q., Jiang X.S., Zhou H.P. (2016). Study of three-dimensional electro-osmotic flow with curved boundary via lattice Boltzmann method. Int. J. Mod. Phys. C.

[22] Su J., Ma L., Ouyang J., Feng C. (2017). Simulations of viscoelastic fluids using a coupled lattice Boltzmann method: Transition states of elastic instabilities. AIP Adv..

[23] Feng Z.G., Michaelides E.E. (2004). The immersed boundary-lattice Boltzmann method for solving fluid-particles interaction problems. J. Comput. Phys..

[24] Takeishi N., Ito H., Kaneko M., Wada S. (2019). Deformation of a red blood cell in a narrow rectangular microchannel. Micromachines.

[25] Ma J., Wang Z., Young J., Lai J.C.S., Tian F.B. (2020). An immersed boundary-lattice Boltzmann method for fluid-structure interaction problems involving viscoelastic fluids and complex geometries. J. Comput. Phys..

[26] Ma Q., Xu Q., Chen Q., Chen Z., Su H., Zhang W. (2019). Lattice Boltzmann model for complex transfer behaviors in porous electrode of all copper redox flow battery with deep eutectic solvent electrolyte. Appl. Therm. Eng..

[27] Qian Y.H., D’Humières D., Lallemand P. (1992). Lattice bgk models for navier-stokes equation. Europhys. Lett..

[28] Guo Z., Zheng C., Shi B. (2002). Discrete lattice effects on the forcing term in the lattice Boltzmann method. Phys. Rev. E.

[29] Guo Z.L., Zheng C.G., Shi B.C. (2002). Non-equilibrium extrapolation method for velocity and pressure boundary conditions in the lattice Boltzmann method. Chin. Phys..

[30] Krüger T. (2012). Computer Simulation Study of Collective Phenomena in Dense Suspensions of Red Blood Cells under Shear.

[31] Snijkers F., D’Avino G., Maffettone P.L., Greco F., Hulsen M.A., Vermant J. (2011). Effect of viscoelasticity on the rotation of a sphere in shear flow. J. Non-Newton Fluid.

[32] Goyal N., Derksen J.J. (2012). Direct simulations of spherical particles sedimenting in viscoelastic fluids. J. Non-Newton Fluid.

[33] Liu B., Lin J., Ku X., Yu Z. (2019). Migration of spherical particles in a confined shear flow of Giesekus fluid. Rheol. Acta.

[34] Kim J.Y., Ahn S.W., Lee S.S., Kim J.M. (2012). Lateral migration and focusing of colloidal particles and DNA molecules under viscoelastic flow. Lab. Chip.

[35] Seo K.W., Kang Y.J., Lee S.J. (2014). Lateral migration and focusing of microspheres in a microchannel flow of viscoelastic fluids. Phys. Fluids.

[36] Wang P., Yu Z., Lin J. (2018). Numerical simulations of particle migration in rectangular channel flow of Giesekus viscoelastic fluids. J. Non-Newton Fluid.

[37] Zhang A., Murch W.L., Einarsson J., Shaqfeh E.S.G. (2020). Lift and drag force on a spherical particle in a viscoelastic shear flow. J. Non-Newton Fluid.

[38] Lu X., Liu C., Hu G., Xuan X. (2017). Particle manipulations in non-Newtonian microfluidics: A review. J. Colloid Interface Sci..

[39] Nam J., Namgung B., Lim C.T., Bae J., Leo H.L., Cho K.S., Kim S. (2015). Microfluidic device for sheathless particle focusing and separation using a viscoelastic fluid. J. Chromatogr. A.

[40] Nam J., Shin Y., Tan J.K., Lim Y.B., Lim C.T., Kim S. (2016). High-throughput malaria parasite separation using a viscoelastic fluid for ultrasensitive PCR detection. Lab. Chip.

